# The effect of emotional motivation on strategy flexibility: the moderating role of task load

**DOI:** 10.3389/fpsyg.2023.1241131

**Published:** 2023-11-27

**Authors:** Yun Wang, Chuanlin Zhu, Dan Zuo, Jingyi Liu, Dianzhi Liu

**Affiliations:** ^1^School of Foreign Languages and Literature, Suzhou University of Science and Technology, Suzhou, Jiangsu, China; ^2^School of Educational Science, Yangzhou University, Yangzhou, Jiangsu, China; ^3^School of Education, Soochow University, Suzhou, Jiangsu, China

**Keywords:** task workload, emotional motivational intensity, emotional motivational direction, cognitive flexibility, moderating role

## Abstract

**Introduction:**

Research has demonstrated that cognitive flexibility is associated with academic achievement, with poorer cognitive flexibility being linked to poorer academic performance. Strategy conversion is an example of cognitive flexibility, which requires individuals to quickly and flexibly switch between strategies depending on the task at hand. Studies have investigated the impact of emotional motivation on cognitive flexibility, with varying results. Furthermore, research has indicated that a high task load increases psychological burden and reduces cognitive flexibility, but few studies have analyzed the impact of task load on the relationship between emotional motivation and cognitive flexibility. This study sought to investigate the effect of emotional motivation on cognitive flexibility based on strategy switching, and the moderating effect of task load.

**Methods:**

Three experiments were conducted. Experiment 1 used forced conversion paradigm with a moderate task load that required participants to estimate tasks using a given strategy. Experiment 2 employed matched conversion paradigm with a high task load, informing participants of the strategies to be used but without any clues, necessitating the selection and execution of appropriate strategies based on the question features. Experiment 3 adopted free conversion paradigm with a relatively low task load, allowing participants to freely choose and execute strategies without any correctness or error criteria.

**Results:**

The intensity and direction of emotional motivation have a complicated and fluctuating impact on the flexibility to utilize strategies. When the task workload is high, the intensity of emotional motivation has a significant impact on strategy utilization flexibility, with low approach motivation being more conducive to flexibility. Conversely, when the task workload is low, the direction of emotional motivation has a greater effect, with high avoidance motivation being more advantageous.

**Discussion:**

This study demonstrated that high workload could bring about a low intensity advantage, while low workload could induce an avoidance direction advantage, suggesting that task load could moderate the impact of emotional motivation on arithmetic strategy utilization flexibility, and avoidance motivation is not always detrimental to cognitive flexibility.

## 1. Introduction

### 1.1 Strategy flexibility

Cognitive flexibility refers to the ability to flexibly switch between tasks, change perspectives or methods, and adjust flexibly to adapt to new changes and rules (Tai et al., 2022). Switching from one task to another involves higher cognitive control, reflected by longer reaction time or more errors, as compared to repeating the same task (Jersild, 1927). Cognitive flexibility is an important indicator of cognitive transfer and inhibitory control. It involves the capacity of individuals to modify their behaviors accurately and promptly in response to changes in their external environment and internal state (Armbruster et al., 2012). This ability is essentially the ability to switch rules, that is, to suppress the prior task and regulate the response required by the present task through top-down processing (Kiesel et al., 2010). Research has demonstrated that cognitive flexibility is associated with academic achievement, with poorer cognitive flexibility being linked to poorer academic performance (Bull and Scerif, 2001).

Estimation is a process of employing certain techniques or rules to approximate the correct answer (Hinault and Lemaire, 2017). Generally, four strategies are utilized for this purpose: Round-Up (RU), which involves rounding up both the numbers to the nearest decade (e.g., 43 × 67 ≈ 50 × 70); Round-Down (RD), which requires rounding down both the numbers to the nearest decade (e.g., 43 × 67 ≈ 40 × 60); Up-Down (UD), which involves rounding the first number up to the nearest decade and the second number down to the nearest decade (e.g., 43 × 67 ≈ 50 × 60); and Down-Up (DU), which involves rounding the first number down to the nearest decade and the second number up to the nearest decade (e.g., 43 × 67 ≈ 40 × 70) (Uittenhove and Lemaire, 2012; Uittenhove et al., 2013). Liu et al. (2021b) investigated the switch cost incurred when transitioning between two strategies: UD and DU. They found that both UD-to-DU and DU-to-UD conversions required costs.

Strategy conversion is an example of cognitive flexibility, which requires individuals to quickly and flexibly switch between strategies depending on the task at hand. Strategy switching is an essential skill for students, and research on learning strategy switching can help improve their understanding and ability to adapt to different situations. Conversion cost is a term used to describe the phenomenon where the reaction time of using the new strategy is slower than that of repeating the previous strategy, or the accuracy of using the new strategy is lower than that of repeating the previous strategy, when switching between two strategies (Lemaire and Lecacheur, 2010). Conversion cost is seen as a significant measure of cognitive flexibility, and the lower the conversion cost, the more powerful the conversion ability, and the better the cognitive flexibility (Kiesel et al., 2010). In this study, strategy flexibility is defined as an individual’s capacity to modify, adjust, and use appropriate strategies to solve two-digit multiplication problems, while strategy switching cost is a specific evaluation indicator of strategy flexibility.

### 1.2 Motivational dimensional model of affect

In the past 50 years, the valence-arousal model has been the primary approach to exploring the cognitive role of emotions. Fredrickson (2001) proposed the broaden-and-build theory of positive emotions, which suggests that positive emotions can heighten cognitive abilities, broaden one’s scope of perception and action, and facilitate cognitive processing (Fredrickson, 1998, 2001; Fredrickson and Branigan, 2005). However, recent studies have cast doubt on this theory. For instance, Phillips et al. (2002) observed that executive control tasks such as the Tower of London and Stroop tasks were inhibited by positive emotions; Levine and Edelstein (2009) found that both positive and negative emotions narrowed the memory range. Subsequently, Gable and Harmon-Jones (2008) proposed the motivational dimensional model of affect, which takes into account the dimension of motivation in addition to valence and arousal. This theory has since gained traction among researchers.

Research conducted by Gable and Harmon-Jones (2008, 2010a,b,c) has revealed that emotion possesses an additional dimension beyond valence and arousal, namely motivation. This emotional motivation dimension model proposes that motivation is composed of two components: intensity and direction. Intensity is further divided into high and low, while direction is divided into approach and avoidance. Thus, emotions can be divided into five categories: high motivational intensity positive emotions (desires and hopes), high motivational intensity negative emotions (disgust, fear, and anxiety), neutral, low motivational intensity positive emotions (pleasure and tranquility), and low motivational intensity negative emotions (sadness and depression). To investigate the impact of emotional motivation on cognitive processing, researchers generally utilize a combination of emotional arousal and cognitive tasks. This involves inducing participants’ emotional states of varying levels of motivation, such as emotional picture arousal, video clip arousal, body posture arousal, and monetary reward arousal, before the participants complete a certain cognitive processing task. The results from different perspectives suggest that high motivational intensity tends to limit the scope of cognitive processing, while low motivational intensity tends to expand the scope of cognitive processing.

In this theory, motivation and valence are not equivalent. For instance, even though anger is an unpleasant sentiment, it can still motivate people to take aggressive action, illustrating its convergent quality. Furthermore, the level of motivation associated with highly arousing emotions, such as humor, is not always strong, thus distinguishing motivation and arousal.

### 1.3 The relation between emotion and strategy utilization

Research has revealed that individual executive function (Ai et al., 2017), age (Geurten and Lemaire, 2017), and emotions (Lallement and Lemaire, 2021) can all influence the use of estimation strategies. Emotions, in particular, have been the focus of much research; for instance, Liu et al. (2021a) conducted an experiment to explore the effect of different emotional states (anger, fear, pleasure, and neutral) on the performance of a two-digit multiplication estimation strategy while controlling arousal. Results indicated that participants responded most quickly when in a pleasurable state, whereas those in a fearful state demonstrated the slowest reaction time, implying that positive emotions can have a beneficial impact on multiplication estimation strategies. Zhu et al. (2021) conducted an investigation into the neurological mechanisms associated with the effect of positive emotions on mathematical estimation strategies, utilizing an implicit emotion priming procedure combined with a dual task design of facial gender judgment and multiplication estimation. Participants were first asked to complete the estimation task, followed by judging the gender of different emotional faces. Unexpectedly, the reaction time was found to be fastest under fear conditions, contradicting prior research on the effect of negative emotions on cognitive processing (Lallement and Lemaire, 2021). The reasons for the discrepancies in the findings of the impact of negative emotions remain uncertain, and whether emotional motivations are involved is yet to be determined. If so, what are the effects of motivational intensity and direction on the strategies employed? This is an area that has yet to be explored.

Studies have investigated the impact of emotional motivation on cognitive flexibility, with results that have been inconsistent. Studies have demonstrated that approach motivation, which is indicative of positive emotions, can bolster cognitive control and reduce the interference effect in response conflict or response inhibition tasks (Van der Stigchel et al., 2011). Conversely, other studies have shown that positive emotions impede cognitive control and generate greater interference effects in response to conflict tasks (Phillips et al., 2002). Martin and Kerns (2011) investigated and found no evidence that positive emotions have an influence on cognitive control. Furthermore, research conducted by Pessoa et al. (2012) revealed that avoidance motivation, which is associated with negative emotions, could either promote, impede, or have no effect on cognitive control. To date, the influence that emotional motivation has on cognitive flexibility has not been fully understood. Moreover, research has revealed that high task load heightens psychological pressure and decreases cognitive flexibility (Wickens, 2008; Fabio et al., 2021), though few studies have examined the effect that task load has on the connection between emotional motivation and cognitive flexibility.

### 1.4 Present study

This study aims to examine how emotional motivation affects strategy use, as well as the possible influence of task load on the relationship between emotional motivation and strategy flexibility. To accomplish this, three experiments were conducted. Experiment 1 used forced conversion paradigm with a moderate task load that required participants to estimate tasks using a given strategy. Experiment 2 employed matched conversion paradigm with a high task load, informing participants of the strategies to be used but without any clues, necessitating the selection and execution of appropriate strategies based on the question features. Lastly, Experiment 3 adopted free conversion paradigm with a relatively low task load, allowing participants to freely choose and execute strategies without any correctness or error criteria.

It is hypothesized that emotional motivation will have a noteworthy impact on strategy utilization flexibility, exhibiting both intensity effect and direction effect (Hypothesis 1). Moreover, the effect of emotional motivation on strategy flexibility is expected to be moderated by task load, with low workloads stimulating flexibility (Hypothesis 2). Examining these questions can reveal the impact of an emotional motivation on cognitive flexibility, and can provide scientific proof for inducing emotional states that are advantageous for improving cognitive flexibility and improving students’ academic performance from the perspective of the emotional motivation.

## 2 Experiment 1: forced-conversion

### 2.1 Methods

#### 2.1.1 Participants

A total of 16 participants were initially calculated using MorePower 6.0 software (*α* = 0.05, test power = 0.95, effect size = 0.25) (Campbell and Thompson, 2012) to improve the power of the statistical test. Sixty college students (21 male) with an average age of 21.62 ± 0.73 years, normal/corrected-to-normal visual acuity, no history of brain trauma or mental illness, who were all right-handed and had not participated in similar studies before, were selected to avoid any potential invalid data or equipment issues during the experiment. All participants signed an informed consent form in accordance with the Declaration of Helsinki (1991) and were rewarded with 50 RMB for their participation. After excluding three participants whose correct rate was less than 70% (the total completion rate of the estimation task and the emotion judgment task), the final sample size was 57.

#### 2.1.2 Materials

The experimental materials employed in this study consisted of 420 two-digit multiplication estimation questions and 210 emotional motivational pictures. The UD (rounding the first number up to the nearest decade, while rounding the second number down to the nearest decade) and DU (rounding the first number down to the nearest decade, while rounding the second number up to the nearest decade) strategies used by Hinault et al. (2014) were utilized in the estimation questions. In addition, seven principles were applied in formulating the multiplication computational estimation problems in order to optimize accuracy: (1) no number had its closest decade equal to 10 or 100; (2) no number had 0 or 5 as its unit digit; (3) numbers were not repeated in the decade or unit; (4) no digits were repeated within numbers; (5) no tie problems were used; (6) the first number was smaller than the second; and (7) the unit of the first number was always below 5 and the unit of the second number was always above 5. The multiplication computational estimation problems and answer choices were presented on the screen with a black background, with the font style of Times New Roman and a font size of 30.

This study selected emotional motivational pictures from the International Emotional Picture System (IAPS) (Lang et al., 2005) and the Internet, consisting of 42 pictures each of delicious food (high approach motivation), beautiful scenery (low approach motivation), threatening scenes (high avoidance motivation), crying people (low avoidance motivation), and neutral images (e.g., a wood, a pen, etc.). All pictures were set to 330 × 240 pixels in size and had consistent transparency and contrast. Five college students were asked to classify the emotions evoked by the pictures before a larger evaluation was conducted. The participants were asked to describe the emotional experience the picture would bring, and 80% agreement was reached. Subsequently, 50 college students (aged 22.65 ± 1.66 years, with normal/corrected-to-normal visual acuity and normal color vision, and all right-handed) were invited to rate the pictures on a 9-point scale from the three dimensions of motivation/arousal/valence (1 representing desperate avoidance, no arousal, and very negative; 9 representing desperate approach, high arousal, and very positive). The participants of the study had no prior experience with similar experiments and did not take part in the formal experiment of this study. To ensure that the food pictures were effective in inducing high approach motivation mood, the participants completed the picture evaluation task around 2 to 3 h after their last meal. The data of motivation, arousal, and valence of the five emotional pictures are presented in Table 1.

**Table 1:** Motivation, arousal, and valence (M ± SD) of different types of emotional motivational pictures (*N* = 50).

	Motivation	Arousal	Valence
High approach	7.02 ± 0.32	5.42 ± 0.48	6.74 ± 0.35
Low approach	6.22 ± 0.36	5.56 ± 0.62	6.60 ± 0.50
Neutral	4.95 ± 0.29	2.34 ± 0.36	5.10 ± 0.44
Low avoidance	2.84 ± 0.38	6.09 ± 0.55	2.22 ± 0.22
High avoidance	1.74 ± 0.20	6.12 ± 0.40	2.13 ± 0.25

M, Mean; SD, standard deviation.

A one-way repeated-measures ANOVA revealed that there were significant differences among the five groups of images in terms of motivation [*F*(4, 240) = 2398.14, *p* < 0.001], arousal [*F*(4, 240) = 507.12, *p* < 0.001], and valence [*F*(4, 240) = 873.48, *p* < 0.001]. *Post-hoc* multiple comparisons revealed no significant difference in arousal (*p* = 0.172) or valence (*p* = 0.063) between high approach and low approach pictures, but a significant difference in motivation (*p* < 0.001). Additionally, no significant difference in arousal (*p* = 0.751) or valence (*p* = 0.253) was observed between high avoidance and low avoidance pictures, although there was a significant difference in motivation (*p* < 0.001). Neutral pictures were found to differ significantly from the other four groups in all three dimensions (*ps* < 0.001). This indicates that the difference in experimental results is primarily due to the different levels of motivation of the various emotions.

#### 2.1.3 Design

This experiment employed a within-subjects design with five emotional motivation types (high approach, low approach, neutral, low avoidance, and high avoidance), two strategy types (UD and DU), and two task types (repetition and switch). The focus of the study was the amount of time taken by the subjects to complete the estimation task.

**Figure 1:**
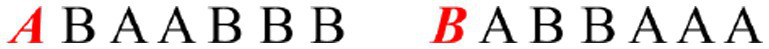
The trial samples in the block (A, UD strategy; B, DU strategy).

If the same strategy is applied twice consecutively, it is referred to as strategy repetition; if different strategies are employed, it is termed a strategy switch. Luwel et al. (2009) utilized an order of AABBAABB, in which A and B represent two distinct strategies. A repetition of strategy A is denoted by A in A**A**, while a switch from strategy A is denoted by A in B**A**. Similarly, strategy B is repeated by B in B**B** and switched by B in A**B**. To prevent the participants, all of whom were college students, from recognizing the basic rules, the study was improved by presenting the switch and repetition trials in a more complex pseudo-random manner, as well as by pairing them with different emotional motivations. Our research developed a sequence of 14 estimation questions, as seen in Figure 1, where the initial strategy in each segment (*A* and *B*, red and italicized in Figure 1) is neither repeated nor switched, thus no records are kept. The remaining 12 questions can be divided into 3 repeated trials and 3 switch trials for the UD strategy, and 3 repeated trials and 3 switch trials for the DU strategy, thus optimizing the utilization of experimental trials. It is hypothesized that the use of a block design will lead to emotional numbness among participants, reducing the differences between different emotional motivations. To avoid this, the experiment was structured with six large blocks, each of which was further divided into 10 small segments with 7 trials each. All trials within each small segment were designed to have the same emotional motivation, thus avoiding an increase in cognitive load for the participants and allowing for more accurate statistical analysis. To eliminate any sequential effects, the order of the six blocks was balanced in terms of motivation. As the first trial in each segment was not considered a repetition or switch, it was not included in the analysis. This study resulted in 90 repetition trials and 90 switch trials for the UD strategy, as well as 90 repetition trials and 90 switch trials for the DU strategy, with 36 repetition trials and 36 switch trials for each of the five emotional motivations.

This study employed a within-subjects design to investigate the effect of emotional motivation on strategy use among three different experimental paradigms, and to further assess the influence of task load. To this end, 60 participants were randomly divided into three groups of 20. A balanced Latin square design was used in each group, with the order of the experiments varying among them (Group A: forced-conversion → matched-conversion → free-conversion; Group B: matched-conversion → free-conversion → forced-conversion; Group C: free-conversion → forced-conversion → matched-conversion). Each subject was required to complete the three experiments with a one-week interval among them.

#### 2.1.4 Procedure

**Figure 2:**
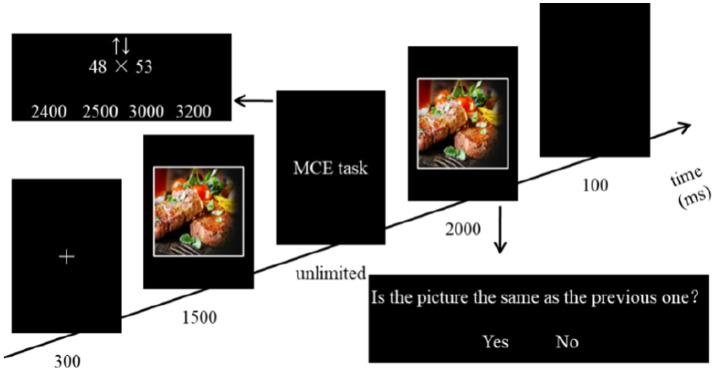
Illustration of one experimental trial in Experiment 1.

Participants were instructed to use the UD and DU strategies before the formal experiment. The procedure consisted of six blocks, each with 70 trials, for a total of 420 trials. As shown in Figure 2, each trial began with a white “+” (300 ms), followed by an emotional motivational picture (1,500 ms), the multiplication computational estimation task (MCE task, unlimited), the picture judgment task (PJ task, 2000 ms), and then a blank (100 ms). Stimuli were presented in the center of the screen. In MCE task, the computational estimation question and strategy cue (i.e., ↑↓ for UD and ↓↑for DU) were presented in the middle of the screen, and four answer choices were presented side by side at the bottom of the screen, corresponding to the response keys of D (left middle finger), F (left index finger), J (right index finger), and K (right middle finger). Two of the choices were the calculated results of the UD and DU strategies, and the other two were the appropriate results (±100/200) of the UD and DU strategies. Participants were asked to identify the correct answer according to the cue. To ensure the occurrence of emotional motivation priming, participants were required to recognize whether the picture was the same as the one that had appeared before the MCE task, and were instructed to press key F (left index finger) if it was and key J (right index finger) if it was not. In both tasks, participants were asked to respond as accurately and quickly as possible. The probability that the correct answer would appear on the left and right was equal.

This study adopted a pseudo-random design, in which the order of the different blocks was randomized across participants to prevent the correct answer from appearing in the same position continuously for more than three times. To ensure that participants fully understood the experiment process, 20 practice trials were provided, with 10 trials for each strategy and four trials for each motivation picture. To control the fatigue effect, all participants were required to rest for 2 min after completing each block before proceeding to the next. Feedback was provided in the practice phase, while no feedback was given in the formal phase. The formal experiment lasted approximately 40 min and was conducted in a soundproof room. Participants were seated approximately 90 cm from a 17-inch screen monitor (resolution 1,024 × 768).

### 2.2 Results

**Figure 3:**
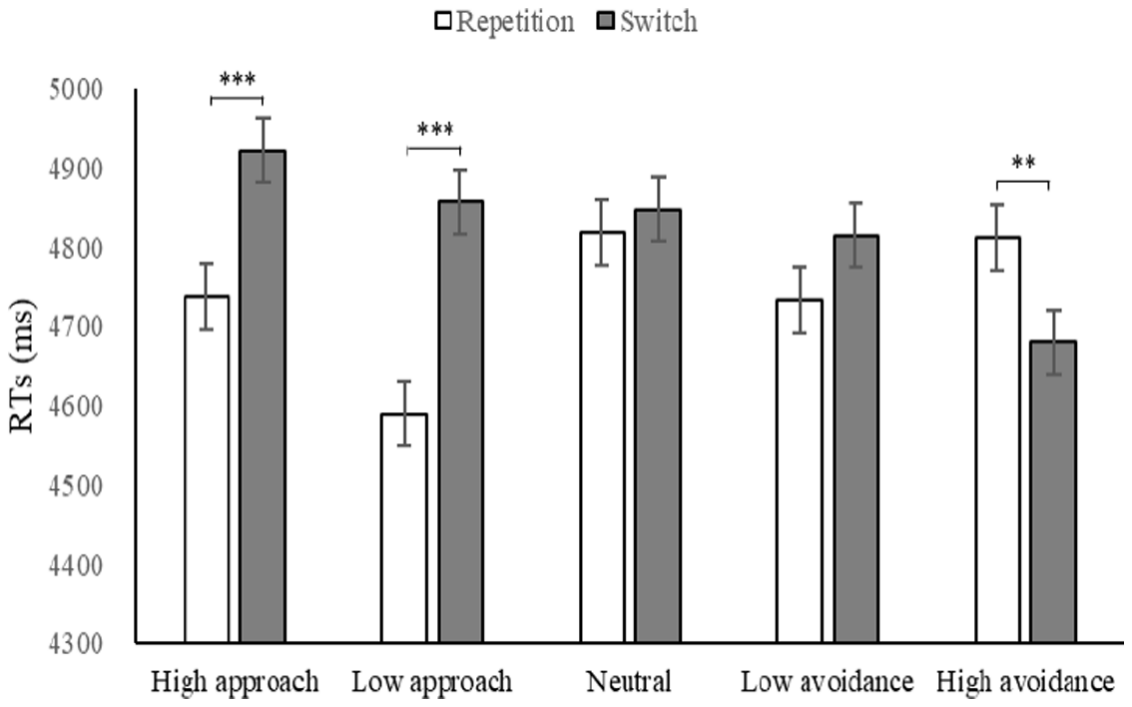
RTs under different motivations in Experiment 1.

Due to the fact that the participants were college students, the estimation task was of a relatively low difficulty, resulting in a correct rate of 97.85% and a ceiling effect. Therefore, the reaction time of completing the estimation task was chosen as the analysis index (Table 2). A repeated-measures ANOVA was conducted to assess the effects of motivation, strategy, and task on reaction time. Results indicated a significant main effect of motivation [*F*(4, 224) = 3.65, *p* = 0.007, *η*2 *p* = 0.06], strategy [*F*(1, 56) = 11.83, *p* = 0.001, *η*2 *p* = 0.17], and task [*F*(1, 56) = 17.49, *p* < 0.001, *η*2 *p* = 0.24]. A significant interaction was observed between motivation and strategy [*F*(4, 224) = 6.55, *p* < 0.001, *η*2 *p* = 0.11], between motivation and task [*F*(4, 224) = 9.19, *p* < 0.001, *η*2 *p* = 0.14], and between strategy and task [*F*(1, 56) = 9.41, *p* = 0.003, *η*2 p = 0.14]. However, the interaction among motivation, strategy, and task was not significant [*F*(4, 224) = 0.77, *p* > 0.05, *η*2 *p* = 0.01]. A Bonferroni-adjusted simple effect analysis revealed that, under high approach and low approach motivation, reaction time for switching tasks was significantly longer than that for repetition tasks (*ps* < 0.001). In contrast, under high avoidance motivation, reaction time for switching tasks was significantly shorter than that for repetition tasks (*p* < 0.001). These findings suggest that emotional motivation has a significant impact on the utilization of strategies. High and low approach motivations were beneficial for the execution of strategy repetition, while high avoidance motivation was favorable for the quicker response to strategy switching (Figure 3).

**Table 2:** RT (reaction time) and strategy switching costs (ms) (*M* ± *SD*) under different conditions in Experiment 1 (*N* = 57).

Motivation	UD repetition	UD switching	DU repetition	DU switching	Costs
High approach	2252.52 ± 531.12	2416.57 ± 564.21	2484.75 ± 564.69	2505.08 ± 536.58	184.37 ± 340.59
Low approach	2225.96 ± 527.50	2392.20 ± 482.85	2363.69 ± 454.47	2464.69 ± 537.78	267.23 ± 448.76
Neutral	2390.01 ± 530.68	2439.03 ± 545.20	2428.37 ± 529.15	2408.06 ± 550.39	28.71 ± 398.37
Low avoidance	2311.53 ± 541.59	2362.29 ± 511.63	2421.47 ± 529.15	2452.13 ± 531.86	81.41 ± 339.62
High avoidance	2364.22 ± 519.00	2346.86 ± 585.19	2446.87 ± 506.56	2332.76 ± 488.65	−131.47 ± 327.24

The cost is calculated by subtracting the repetition reaction time of both the UD and DU from the switching reaction time of both the UD and DU.

**Figure 4:**
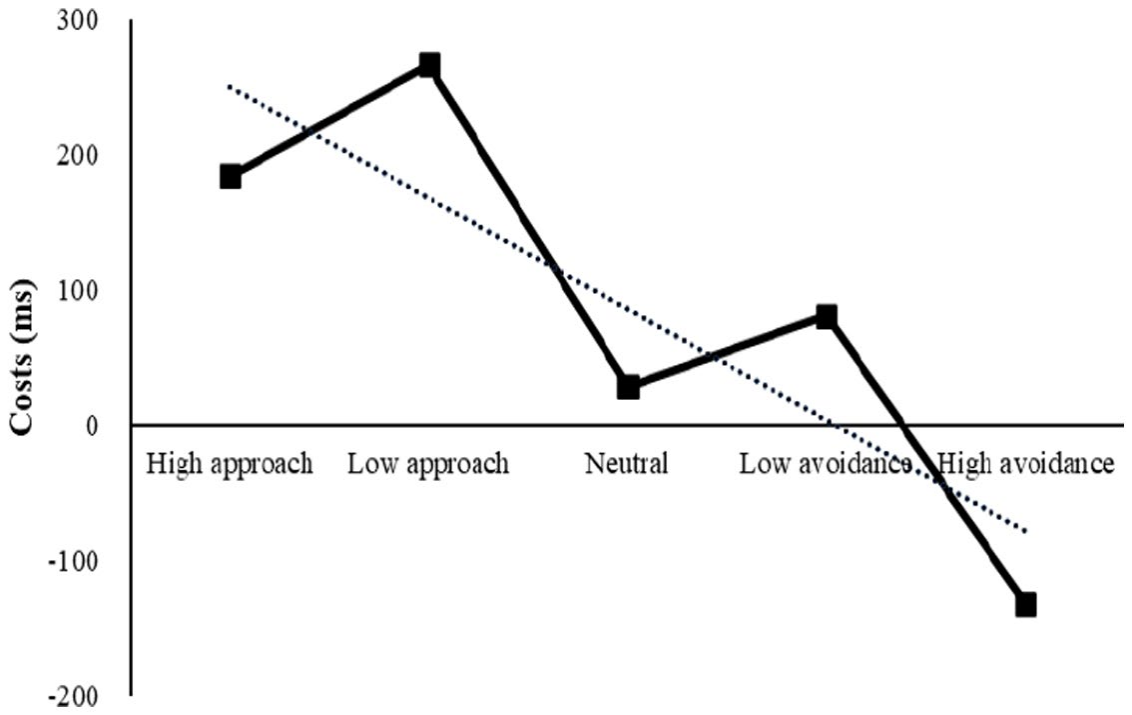
Costs under different motivations in Experiment 1 and the trendline represents the changing trend of switching cost.

A repeated-measures ANOVA was conducted to evaluate the strategy switching cost under different motivations. The results revealed a significant main effect, *F*(4, 224) = 9.19, *p* < 0.001, *η*2 *p* = 0.14. *Post-hoc* pairwise comparisons showed that the cost under high avoidance was the lowest, significantly lower than the cost under other motivations (*ps* < 0.01). This suggests that high avoidance is the most effective motivation to enhance the flexibility of strategy utilization. Additionally, there was a significant difference between high avoidance and low avoidance motivation intensity (*p* = 0.003), but not between high approach and low approach motivation intensity (*p* = 0.147), indicating that the intensity effect was partially significant. The significance was found between high approach and high avoidance motivation direction (*p* < 0.001), and between low approach and low avoidance motivation direction (*p* = 0.007), indicating that the direction effect was significant. Figure 4 demonstrated a downward trend in the transition from approach motivation to avoidance motivation, as evidenced by the changing trend of strategy switching cost.

The results of Experiment 1 showed a partial verification of Hypothesis 1. Specifically, the intensity effect of emotional motivation was only partially significant, while the direction effect was entirely significant. The intensity effect was demonstrated through avoidance motivation, as greater intensity resulted in better flexibility. This finding diverged from Gable and Harmon-Jones’ research (2010a), which proposed that high intensity impeded flexibility. However, from an evolutionary standpoint, this result was more realistic. It was suggested that individuals require quick decisions in emergency situations in order to mobilize attention resources and raise vigilance and flexibility within a short time (Fox et al., 2002). It was clearly evident that the costs under high and low avoidance motivations were lower than those under high and low approach motivations, indicating that avoidance motivation significantly enhanced flexibility. This is an unprecedented finding in this study and has not been reported in preceding studies. This may be attributed to the inherent characteristics of avoidance motivation and task switching. The avoidance motivation model suggests that withdrawal from the current stimulus is an inherent attribute of this type of motivation, while switching task is characterized by withdrawal from the original task and completion of the present task. When avoidance motivation is successfully activated, the withdrawal effect of inhibiting the current stimulus is automatically triggered, and switching tasks can be completed quickly. However, low approach was not conducive to switching, which is inconsistent with the emotional motivation model (Gable and Harmon-Jones, 2008, 2010b). It is possible that this discrepancy is related to the task load of the experimental paradigm. Experiment 1 used the forced-switching paradigm, which provided strategy cues and only required participants to execute the specified strategy, while Experiment 2 used the matched-switching paradigm that removed strategy cues and necessitated participants to search for the relevant strategies to match the characteristics of the estimation tasks. This allowed for further exploration of the moderating role of task load between emotional motivation and strategy flexibility.

## 3 Experiment 2: matched-conversion

### 3.1 Methods

#### 3.1.1 Participants

The participants who took part in Experiment 2 were the same as those in Experiment 1.

#### 3.1.2 Materials

The materials used for this experiment were the same as those in Experiment 1.

#### 3.1.3 Design

The design was identical to that of Experiment 1.

#### 3.1.4 Procedure

**Figure 5:**
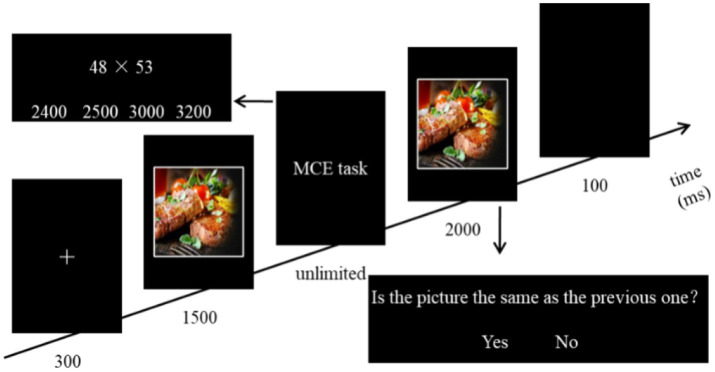
Flowchart of single trial in Experiment 2.

Before the experiment, the participants were informed of the meaning of matching strategies: if the unit digit of the multiplicand in the two multipliers is greater than 5 and the unit digit of the multiplier is less than 5, then the UD strategy should be used; otherwise, the DU strategy should be used. They were also informed that they must use the matching strategies, and that any incorrect strategies would result in incorrect results. Unlike Experiment 1, no strategy cues (arrows) were presented to the participants in Experiment 2, thus requiring them to search for the appropriate strategies, resulting in a higher workload (Figure 5).

### 3.2 Results

**Figure 6:**
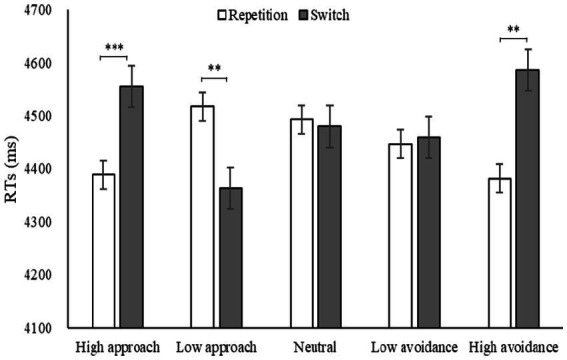
RTs under different motivations in Experiment 2.

Just like Experiment 1, the accuracy was 98.39%, a level that reached the ceiling effect, so the only data that could be analyzed was the reaction time (Table 3). A 5 × 2 × 2 repeated-measures ANOVA of motivation, strategy, and task was conducted with reaction time as the dependent variable. The results revealed a significant main effect of strategy, *F*(1, 56) = 24.92, *p* < 0.001, *η*2 *p* = 0.31, and a marginally significant main effect of task, *F*(1, 56) = 3.84, *p* = 0.055, *η*2 *p* = 0.06. The main effect of motivation was not significant, *F*(4, 224) = 0.66, *p* > 0.05, *η*2 *p* = 0.01. Significant interactions existed between motivation and strategy [*F*(4, 224) = 3.18, *p* = 0.014, *η*2 p = 0.05], between motivation and task [*F*(4, 224) = 10.26, *p* < 0.001, *η*2 *p* = 0.16], and between strategy and task [*F*(1, 56) = 73.84, *p* < 0.001, *η*2 *p* = 0.56]. However, the interaction among motivation, strategy, and task was not significant, *F*(4, 224) = 1.48, *p* > 0.05, *η*2 *p* = 0.03. However, post-hoc pairwise comparisons showed that switching RTs were significantly longer than repeating RTs under both high approach (*p* < 0.001) and high avoidance (*p* = 0.002). Conversely, under low approach motivation, switching RTs were significantly shorter than repeating RTs (*p* = 0.002). These findings suggest that high approach motivation and high avoidance motivation favor repetition tasks, whereas low approach motivation favors conversion tasks (Figure 6).

**Table 3:** RT and strategy switching costs (*M* ± *SD*) under different conditions in Experiment 2 (*N* = 57).

Motivation	UD repetition	UD switching	DU repetition	DU switching	Costs
High approach	2073.88 ± 449.52	2227.73 ± 476.25	2314.74 ± 561.83	2326.67 ± 572.71	165.78 ± 271.90
Low approach	2169.29 ± 486.63	2140.02 ± 424.84	2347.73 ± 602.67	2222.31 ± 527.38	−154.69 ± 360.81
Neutral	2176.25 ± 483.05	2250.54 ± 506.90	2316.44 ± 510.37	2228.80 ± 501.74	−13.34 ± 298.14
Low avoidance	2129.85 ± 447.12	2183.10 ± 483.50	2316.51 ± 546.19	2275.76 ± 520.09	12.50 ± 314.44
High avoidance	2080.42 ± 456.32	2292.65 ± 539.82	2301.13 ± 525.51	2293.68 ± 504.98	204.79 ± 463.30

**Figure 7:**
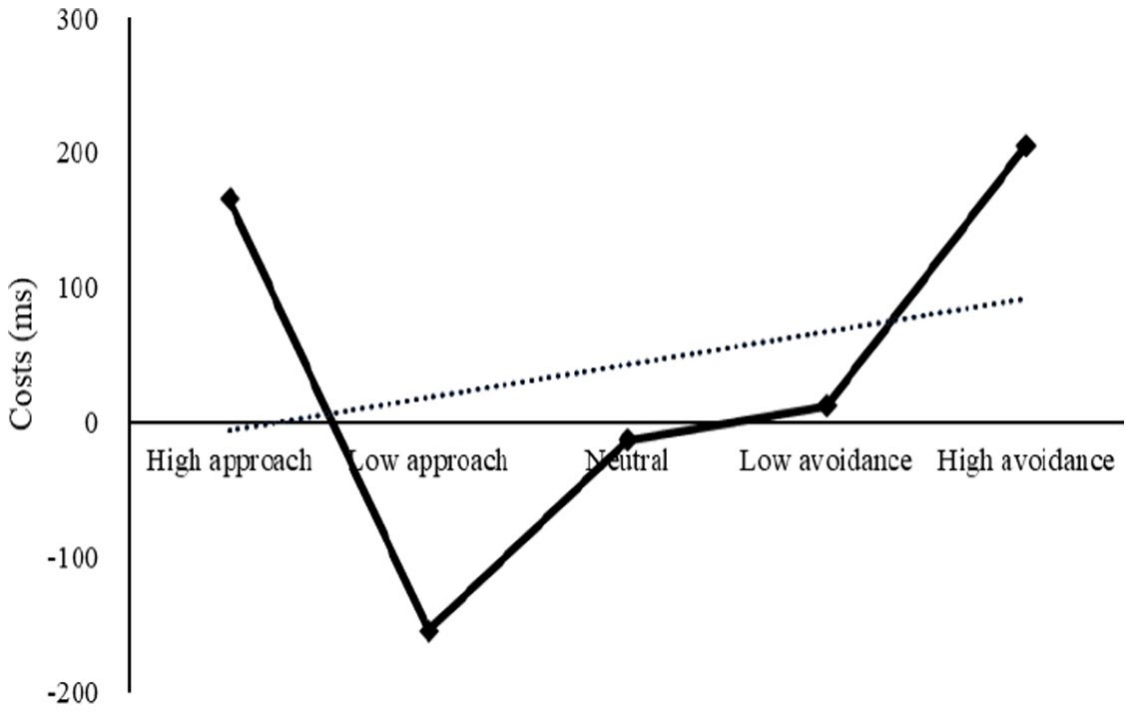
Costs under different motivations in Experiment 2.

An analysis of the changing trend of strategy switching costs revealed a W-shaped pattern, with a first sharp decline followed by a tortuous rise (Figure 7). A repeated-measures ANOVA was conducted to evaluate the effect of motivation on strategy utilization flexibility, with the results showing a significant main effect of motivation, *F*(4, 224) = 10.26, *p* < 0.001, *η*2 *p* = 0.16. Post-hoc pairwise comparisons further revealed that costs under low approach motivation were significantly lower than those under other motivations (*ps* < 0.05). Additionally, a significant difference was found between high approach and low approach (*p* < 0.001), as well as between high and low avoidance (*p* = 0.008), indicating that the intensity effect was significant. Furthermore, a significant difference was observed between low approach and low avoidance (*p* = 0.006), while no significant difference existed between high approach and high avoidance (*p* = 0.551). Thus, the direction effect was partially significant.

Experiment 2 demonstrated that emotional motivation had a significant effect on the flexibility of strategy utilization under the matched-conversion paradigm, although the direction effect was only partially significant. This partially verified Hypothesis 1. It was found that intensity had a significant impact on both approach and avoidance motivation: high intensity motivation was not beneficial for task switching and even caused a greater switch loss, whereas low intensity motivation facilitated the task switch and reduced switch loss. This finding was in agreement with the results of previous studies, which showed a directional effect only present in cases of low-intensity motivations, with a significant low approach advantage aiding conversion, as predicted by the motivational dimensional model (Gable and Harmon-Jones, 2008, 2010a,b). It is noteworthy that the traits associated with high approach are comparable to those of Experiment 1, and both are detrimental to flexibility. In Experiment 2, the results of low approach and high avoidance were found to be in stark contrast to those of Experiment 1, suggesting that task workload had an effect on the relationship between emotional motivation and strategy flexibility. This partially supports Hypothesis 2. To further explore the influence of task workload and increase the ecological validity of the study, Experiment 3 will employ the free-conversion paradigm. This experiment will reduce the participants’ task workload and examine the impact of emotional motivation on strategy application in a more natural setting.

## 4 Experiment 3: free-conversion

### 4.1 Methods

#### 4.1.1 Participants

Experiment 3 had fewer effective subjects than Experiments 1 and 2, which was due to the task requirements. This experiment was conducted to assess the effect of emotional motivation on flexibility in a natural state, regardless of the accuracy of the strategy application. Thus, 15 subjects with an accuracy rate below 70% were excluded, resulting in a total of 45 effective subjects.

#### 4.1.2 Materials

The materials used in Experiment 3 were identical to those used in Experiments 1 and 2.

#### 4.1.3 Design

Experiment 3 followed the same design as Experiments 1 and 2.

#### 4.1.4 Procedure

In Experiment 3, the subjects were free to choose either a UD or a DU strategy to complete the estimation task without any restrictions on the correctness or error of the strategy application. This was in contrast to Experiment 2, in which the correctness of the strategy application was explicitly demanded. Participants were allowed to select either a UD strategy or a DU strategy to complete the estimation task; however, only the trials that had corresponding question characteristics and strategy types were judged as correct and included in the final data analysis.

### 4.2 Results

The purpose of this experiment was to examine the impact of emotional motivation on flexibility in a natural setting, with no accuracy prerequisite; thus, reaction time was selected as the only index for analysis (Table 4).

**Table 4:** RT and strategy switching costs (*M* ± *SD*) under different conditions in Experiment 3 (*N* = 45).

Motivation	UD repetition	UD switching	DU repetition	DU switching	Costs
High approach	2022.16 + 442.73	2225.46 ± 586.94	2442.27 ± 1045.70	2370.99 ± 757.03	108.21 ± 709.90
Low approach	2029.57 ± 520.62	2121.08 ± 533.48	2283.00 ± 612.71	2297.08 ± 624.43	102.06 ± 341.42
Neutral	2161.77 ± 497.72	2195.17 ± 620.51	2326.44 ± 740.65	2313.80 ± 965.48	−84.89 ± 331.09
Low avoidance	2073.74 ± 532.51	2136.31 ± 554.91	2388.00 ± 922.98	2302.05 ± 775.60	−38.54 ± 383.15
High avoidance	2154.16 ± 555.78	2155.74 ± 597.51	2318.24 ± 648.31	2226.34 ± 618.55	−131.10 ± 368.72

**Figure 8:**
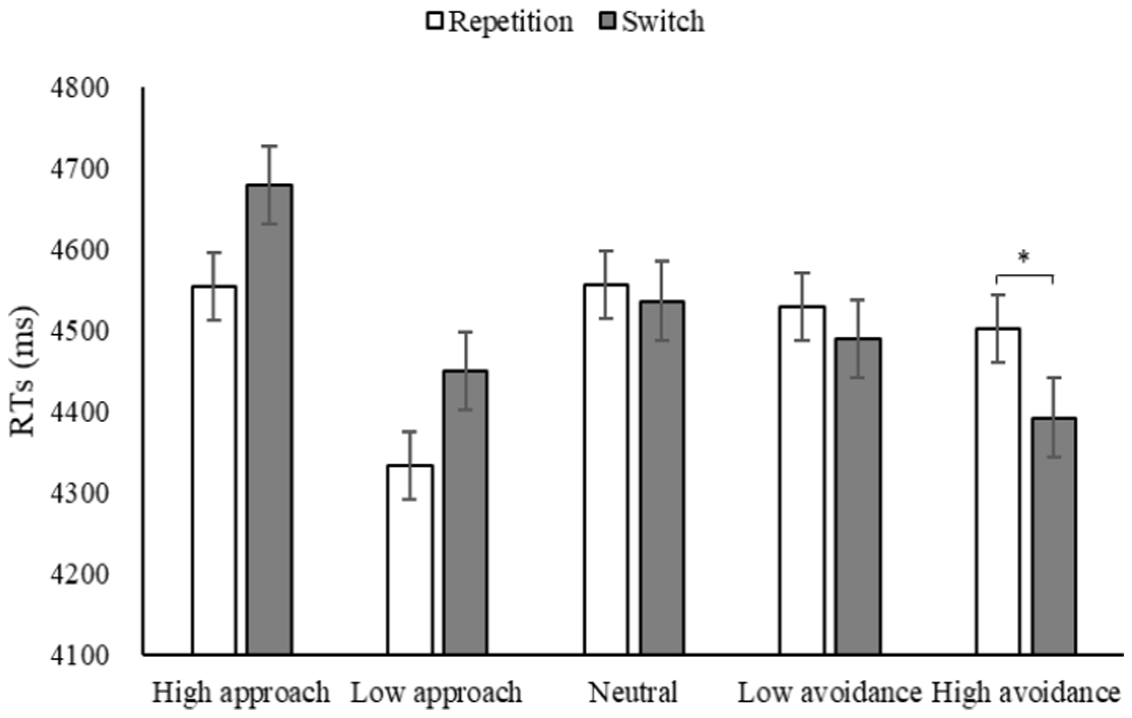
RTs under different motivations in Experiment 3 (*p < 0.05).

A repeated-measures ANOVA was conducted to examine the effects of motivation, strategy, and task. The main effect of motivation was found to be significant, *F*(4, 176) = 4.80, *p* = 0.001, *η*2 *p* = 0.10, indicating that emotional motivation had an impact on strategy application. The main effect of strategy was also significant, *F*(1, 44) = 38.37, *p* < 0.001, *η*2 *p* = 0.47, while the main effect of task type was not, *F*(1, 44) = 0.23, *p* > 0.05, *η*2 *p* = 0.01. A noteworthy interaction was found between motivation and strategy [*F*(4, 176) = 4.05, *p* = 0.004, *η*2 *p* = 0.08], between motivation and task [*F*(4, 176) = 6.31, *p* < 0.001, *η*2 *p* = 0.13], as well as between strategy and task [*F*(1, 44) = 32.91, *p* < 0.001, *η*2 *p* = 0.43]. However, the interaction among the three variables was not significant, *F*(4, 176) = 1.29, *p* > 0.05, *η*2 *p* = 0.03. *Post-hoc* pairwise comparisons revealed that switching RTs were significantly shorter than repetition RTs when participants were under high avoidance motivation (*p* = 0.020), while marginally significantly longer than repetition RTs when under low approach motivation (*p* = 0.051). This indicates that the free conversion paradigm facilitated the switch task when participants were under high avoidance, while hindering it when under low approach (Figure 8).

**Figure 9:**
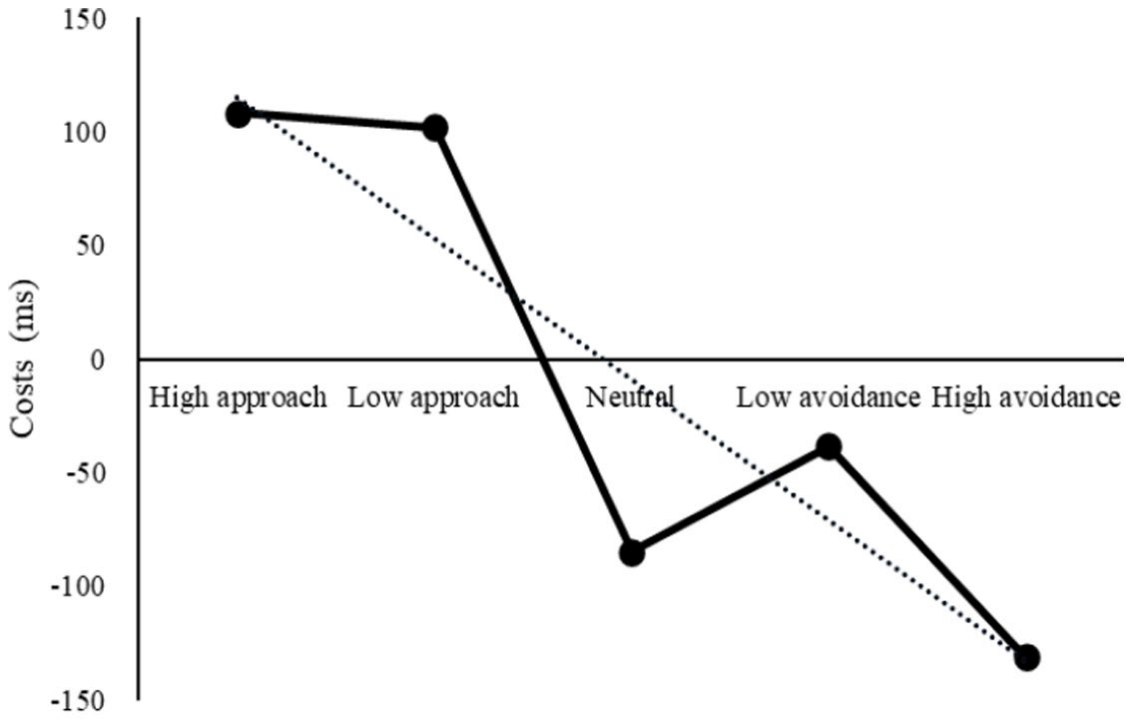
Costs under different motivations in Experiment 3.

An analysis of the data from Experiment 3 (Figure 9) showed a decrease in strategy costs from high approach to high avoidance, which was similar to the results of Experiment 1. A repeated-measures ANOVA revealed a statistically significant main effect of motivation, *F*(4, 176) = 6.31, *p* < 0.001, *η*2 *p* = 0.13. Results indicated that high avoidance was the most effective for flexibility, with switch costs significantly lower than those under high approach and low approach (*ps* = 0.001). No significant differences were found between high approach and low approach (*p* = 0.291) or between high avoidance and low avoidance (*p* = 0.146) in terms of motivation intensity. However, significant differences were observed between high approach and high avoidance (*p* = 0.001), and between low approach and low avoidance (*p* = 0.046) in terms of motivation direction.

Results of Experiment 3 showed that, while the intensity effect of emotional motivation on the flexibility of strategy application was absent under the free-conversion paradigm, the direction effect remained significant. This was in partial agreement with the outcome of Experiment 1, which had revealed the same direction effect. Since the task load in Experiments 1 and 3 was lower than that in Experiment 2, this suggested that low workload can have a similar impact on cognitive flexibility, with avoidance direction being more advantageous. Consequently, Hypothesis 1 was partially validated. In combination with the results from Experiments 1 and 2, it can be concluded that task workload can influence the association between emotional motivation and flexibility. When the task load is reduced, the potential of low intensity motivation to enhance cognitive flexibility diminishes, and the effect of low motivation is related to the degree of the task workload required. This partially confirms Hypothesis 2.

## 5 A comparison of the intensity and direction effects among Experiments 1, 2, and 3

**Figure 10:**
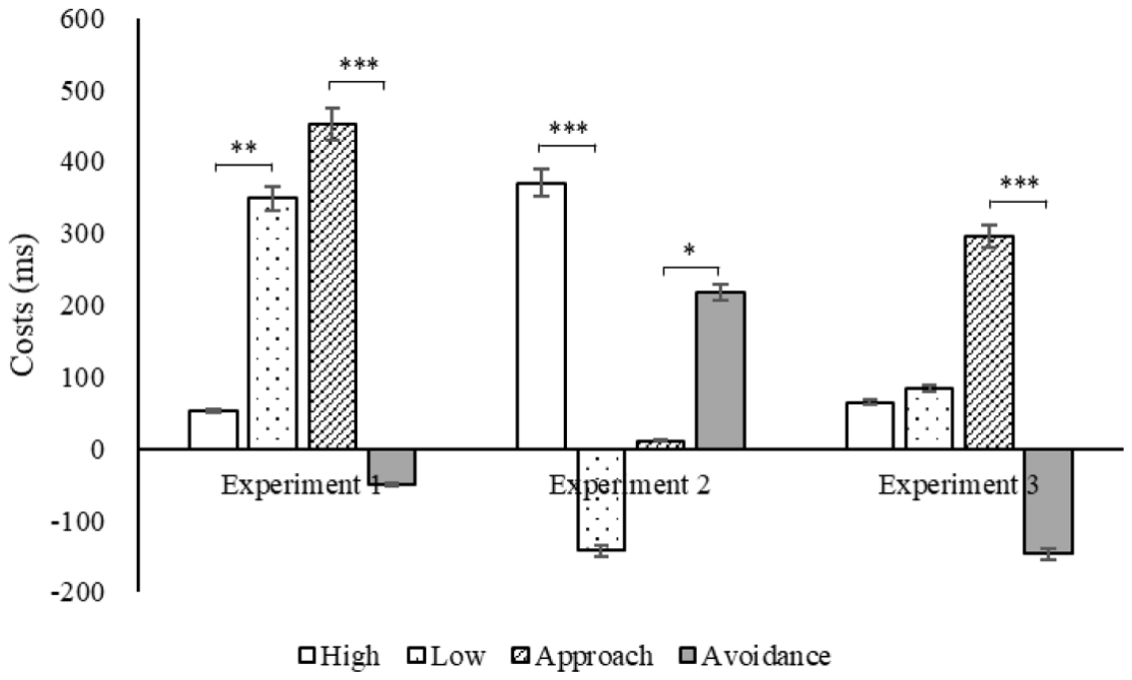
Switching costs under different conditions in three experiments (*p < 0.05, **p < 0.01, ***p < 0.001).

The results of a paired-samples *t*-test on the switching costs of emotional motivation in two dimensions (intensity and direction) in three experiments are shown in Table 5 and Figure 10.

**Table 5:** The results of a paired-samples *t*-test on the intensity and direction effects in different EXP. (experiments).

Exp.	Intensity				Direction			
	High	Low	*p*	Cohen’s *d*	Approach	Avoidance	*p*	Cohen’s *d*
1	52.90 ± 369.04	348.63 ± 618.64	0.003	0.76	451.60 ± 673.75	−50.07 ± 424.67	<0.001	0.69
2	370.56 ± 579.30	−142.19 ± 509.57	<0.001	0.72	11.08 ± 463.76	217.28 ± 590.92	−0.020	0.28
3	64.70 ± 535.20	84.47 ± 582.22	0.856	0.10	295.64 ± 498.00	−146.48 ± 530.78	<0.001	0.67

Table 5 reveals that, with the exception of the intensity effect in Experiment 3, all the intensity and direction effects were statistically significant, demonstrating that emotional motivation had a significant impact on strategy flexibility irrespective of the paradigm. Analysis of a repeated-measures ANOVA on high motivation, low motivation, approach motivation, and avoidance motivation as independent variables with the conversion cost as the dependent variable revealed a significant main effect of high motivation [*F*(2, 86) = 4.23, *p* = 0.018, *η*2 *p* = 0.09], with the conversion cost of Experiments 1 and 3 being significantly lower than that of Experiment 2 (*p* = 0.010, *p* = 0.032), but no significant difference between the first two (*p* = 0.976). Similarly, the main effect of low motivation was found to be significant [*F*(2, 88) = 7.83, *p* = 0.001, *η*2 *p* = 0.15], with the conversion cost of Experiment 2 being significantly lower than that of Experiment 1 (*p* < 0.001), but no significant difference between the other two paradigms (*ps* > 0.05). In addition, the main effect of approach motivation was significant [*F*(2, 88) = 9.80, *p* < 0.001, *η*2 *p* = 0.18], with the conversion cost of Experiment 2 being significantly lower than those of Experiments 1 and 3 (*p* < 0.001, *p* = 0.005), but no significant difference between the latter two (*p* = 0.199). Lastly, the main effect of avoidance motivation was found to be significant [*F*(2, 88) = 6.77, *p* = 0.002, *η*2 *p* = 0.13], with the conversion cost of Experiments 1 and 3 being significantly lower than that of Experiment 2 (*p* = 0.004, *p* = 0.005), but no significant difference between the first two (*p* = 0.495). Results suggest that the intensity and direction of emotional motivation have a considerable effect on strategy flexibility, contingent upon the task setting. Specifically, task load appears to have a regulatory effect on emotional motivation and flexibility, with low motivation having the most significant effect on cognitive flexibility when task load is high, and avoidance motivation having the most influence when task load is low.

## 6 General discussion

The findings of Experiment 1, which included three components: strategy execution, result accuracy, and time pressure, indicated that the motivational intensity effect was partially significant, while the direction effect was significant. Experiment 2, which did not contain any cues, comprised four elements: strategy selection, strategy enactment, result accuracy, and time pressure. The results of this experiment indicated that the intensity effect was significant and the direction effect was partially significant. Experiment 3, which did not necessitate the accuracy of the results, encompassed two tasks: strategy selection and strategy implementation. The results of this experiment revealed that the intensity effect was no longer present, but the direction effect was significant. These outcomes suggest that the influence of emotional motivation on flexibility is affected by the task workload, which can actively modulate the association between them.

### 6.1 The intensity of motivation and the adaptability of strategy implementation

This study partially concurs with the emotional motivational model that proposes that high motivation tends to restrict cognitive processing and impede cognitive flexibility (Gable and Harmon-Jones, 2010b). Our findings revealed that high approach motivation does not have a positive effect on the completion of switching tasks, regardless of the switching paradigm; this is in line with the emotional motivation model proposed by Gable et al. In contrast, the impact of high avoidance motivation on cognitive flexibility is contingent upon the task workload. When the task workload is low, the negative effects of high avoidance motivation are reduced, and its influence on cognitive flexibility is even reversed, suggesting that task switching with low workload can improve cognitive flexibility when driven by high avoidance motivation.

As for low intensity motivation, Kanske and Kotz (2010) proposed the Limited Attention Resources Theory, which suggests that high motivation can aid in concentrating cognitive resources on the current goal, and low motivation can enhance perceptual breadth and response flexibility. However, this study revealed that the positive effect of low motivation intensity on cognitive flexibility is only valid when task workloads are heavy. It may be that when task workloads are reduced, the extra attention resources can cause a distraction, thus diminishing its positive effect on flexibility. Thus, it is evident that task workload is a crucial factor in this regard.

### 6.2 The direction of motivation and flexibility to apply strategies

Previous studies have shown that the direction of motivation has a considerable effect on cognitive flexibility, with avoidance motivation having a particularly detrimental effect (Gable et al., 2016, 2022). Nonetheless, the experiments of this study demonstrated that, when the task workload is adjusted to a specific level, avoidance motivation can be used to efficiently complete the tasks which require flexible conversion, implying that the influence of emotional motivation on flexibility should not be overgeneralized.

Experiments 1 and 3 demonstrated that avoidance direction motivation (low avoidance and high avoidance) had a significant positive effect on flexibility when the task workload was reduced by providing strategy cues to the subjects or not requiring correctness of the results. This finding was similar to that of Roskes et al. (2012), who discovered that the difficulty of the task itself would influence the correlation between avoidance motivation and cognitive processing. When the task structure was favorable, the flexibility under avoidance motivation increased (Rietzschel et al., 2007). This has caused avoidance motivation to be referred to as survival motivation (Oertig et al., 2013), as the negative emotion associated with avoidance motivation can motivate individuals to be more alert and flexible, allowing them to quickly complete tasks and evade unpleasant stimuli. This research further confirms the positive role of avoidance motivation under certain conditions, providing empirical evidence to back this up from the viewpoint of workload.

The results of this study suggest that emotional motivation can have an impact on strategy utilization flexibility, both in terms of its intensity and direction. Additionally, task workload was found to have a dynamic regulatory effect on the relationship between them. Hypotheses 1 and 2 were partially confirmed; however, caution should be taken when interpreting the results, since it is important to consider that the majority of existing studies classify sadness as a low approach motivation (Gable and Harmon-Jones, 2010a; Gable et al., 2016) which is distinct from the low avoidance motivation that was rated in this study. Experiments 1, 2, and 3 all revealed significant distinctions between low approach and low avoidance, yet further research is required to investigate this further. Additionally, it is noteworthy that the International Affective Picture System (IAPS) has been subject to criticism due to its systematic variations in emotional value and methodological issues. Specifically, the standardized ratings may not accurately reflect the range of emotions experienced in the real world, and the selection of images could be affected by subjectivity and cultural bias (Lang and Bradley, 2007). Therefore, we suggest that newer systems, such as the Chinese Affective Picture System (CAPS) (Bai et al., 2005), be taken into account for future research.

## 7 Conclusion

The results demonstrated that emotional motivation had an effect on strategy flexibility, and the impact of emotional motivation on strategy flexibility could be moderated by task workload. To be specific, when the task load was high (Experiment 2), low intensity of motivation was advantageous for cognitive flexibility, and approach motivation was beneficial for cognitive flexibility. In Experiment 1, when the task load was at a moderate level, high intensity and avoidance motivation were beneficial for strategy flexibility. In Experiment 3, with lower task load, the intensity effect was absent; however, avoidance motivation was significantly advantageous for strategy flexibility.

It is evident that high approach motivation consistently resulted in unfavorable cognitive flexibility in all paradigms. However, the effects of low approach and high avoidance motivation were contingent on the task load; when the load was high, low approach induced flexibility while high avoidance hindered it, and when the load was low, the situation was reversed, with high avoidance fostering flexibility and low approach impeding it.

## The educational relevance and implications statement

These findings establish a scientific foundation for inducing emotional states that foster cognitive flexibility during authentic teaching scenarios, thereby enhancing students’ academic achievements.

## Data availability statement

The original contributions presented in the study are included in the article/supplementary material, further inquiries can be directed to the corresponding author.

## Ethics statement

The studies involving humans were approved by Suzhou University of Science and Technology. The studies were conducted in accordance with the local legislation and institutional requirements. The participants provided their written informed consent to participate in this study.

## Author contributions

YW, JL, and DZ designed the study and were responsible for the data collection, conducted the analyses, and interpreted the results. WY took the lead in the writing process. CZ and DZ helped draft the manuscript. DZ participated in the data collection and provided critical feedback for the manuscript. CZ helped with the literature review and references. All authors contributed to the article and approved the submitted version.
